# Pre-existing oncohematological disease in kidney transplant recipients: impact on graft survival, acute rejection, and long-term clinical outcomes

**DOI:** 10.3389/fimmu.2025.1629521

**Published:** 2025-08-06

**Authors:** Alberto Mella, Roberta Clari, Valeria Deiana, Roberta Giraudi, Gloria Giovinazzo, Ester Gallo, Caterina Dolla, Antonio Lavacca, Ana Maria Manzione, Fabrizio Fop, Anna Allesina, Federica Cavallo, Sara Bringhen, Dario Ferrero, Roberto Mina, Corrado Tarella, Benedetto Bruno, Filippo Mariano, Luigi Biancone

**Affiliations:** ^1^ Renal Transplantation Center “A. Vercellone,” Division of Nephrology, Dialysis and Transplantation, Città Della Salute e Della Scienza Hospital, Turin, Italy; ^2^ Department of Medical Sciences, University of Turin, Turin, Italy; ^3^ Nephrology Unit, ASL TO5, Chieri, Italy; ^4^ Division of Hematology, Department of Molecular Biotechnology and Health Sciences, University of Torino, Azienda Ospedaliero Universitaria (A.O.U) Città della Salute e della Scienza di Torino, Turin, Italy; ^5^ Hemato-Oncology Division, Istituto Europeo di Oncologia (IEO), European Institute of Oncology IRCCS, Milan, Italy; ^6^ Dipartimento Scienze Salute, University of Milan, Milan, Italy

**Keywords:** oncohematological diseases, kidney transplant, graft survival, clinical outcomes, acute rejection

## Abstract

**Introduction:**

Oncohematological disorders are heterogeneous conditions that present significant challenges in management prior to transplantation. Data about rejection risk, disease recurrence, eligibility criteria, and requested remission time before kidney transplant (KT) are still lacking.

**Methods:**

All KTRs between January 1, 2000, and March 31, 2023 (n = 2871) were analyzed. All patients with an oncohematological disease (hematological cohort, including plasma cell dyscrasias [PCDs], acute leukemia, high-grade lymphoma/post-transplant lymphoproliferative disorders [PTLDs], myeloproliferative neoplasms [MPNs], myelodysplastic/myeloproliferative neoplasms [MDS/MPNs], and genetic/AA amyloidosis) were matched 1:2 by age at transplant, gender, type of dialysis, and eGFR at transplant with KTRs without a history of hematological disease (control cohort). Primary endpoints were death-censored graft survival and the risk of rejection. Secondary endpoints included the risk of hematological disease recurrence and infection, patient survival rates, and graft function.

**Results:**

Thirty out of 2871 patients (1.04%) receiving 31/3019 KTs have a pre-existing oncohematological disease (hematological cohort): 7/30 (23.3%) PCDs, 4/30 (13.3%) acute leukemia, 8/30 (26.7%) high-grade lymphomas/PTLDs, 4/30 (13.3%) MPNs, 2/30 (6.7%) MDS/MPNs, and 5/30 (16.7%) AA/familiar amyloidosis. Patients were transplanted at a median time of 5 (PCDs), 11.8 (acute leukemia), 12.3 (high-grade lymphomas/PTLDs), 8.5 (MPNs), 3.6 (MDS/MPNs), and 3.5 years (amyloidosis) after achieving disease remission (or stable disease in smoldering myeloma, MPNs, and MDS/MPNs). Comparing hematological and control cohorts, no differences were observed in patient and graft survival or post-transplant complications, including acute rejections. Results are superimposable also without considering the three patients who underwent living KTs from the same donor as the bone marrow transplant. Hematological disease relapses were observed in 2/30 (6.6%), including a light-chain deposition and a Castleman disease, both of which were successfully treated with chemotherapy without allograft dysfunction.

**Conclusions:**

Favorable long-term transplant and clinical outcomes were achieved in patients with various pre-existing oncological and hematological disorders. These patients should not be denied KT after a well-documented stable disease. In this context, a multidisciplinary approach is crucial for establishing standardized pre- and post-transplant monitoring protocols and achieving optimal graft and patient outcomes.

## 1 Introduction

Oncohematological disorders encompass a range of diseases with distinct incidence, presentation, and outcomes, which may also contribute to or be associated with end-stage renal disease (ESRD). Not surprisingly, given the prolonged life expectancy and the improvement of therapeutic armamentarium, these conditions are more frequently observed and well-treated, highlighting challenging questions in pre-transplant settings.

Unfortunately, Literature data about rejection risk and disease recurrence of patients with a previous history of oncohematological diseases before kidney transplant (KT) are still limited. International guidelines vary in their eligibility criteria and requested remission times (1–5). We have previously reported our favorable experience in kidney transplant recipients (KTRs) with a previous history of monoclonal gammopathy of undetermined significance (MGUS) (6); in this study, we now focus our attention on all subjects with a pre-existing hematological disease, including plasma cell dyscrasias (PCDs) (i.e., smoldering [SMM] or multiple myeloma [MM], light chain deposition disease [LCDD], AL amyloidosis), acute leukemia. high-grade lymphoma (including also previous post-transplant lymphoproliferative disorders [PTLD]), myeloproliferative neoplasms (MPNs), myelodysplastic/myeloproliferative neoplasms (MDS/MPN), and genetic/AA amyloidosis.

## 2 Methods

### 2.1 Study population and data collection

The study included all the KTRs performed at the Turin University Renal Transplant Center “A. Vercellone” from January 2000 to March 2023. Patients with a pre-transplant oncohematological disease, classified according to the WHO 5^th^ criteria (7–9) (hematological cohort), were included in the analysis.

All patients were initially managed by the Renal Transplant Center (Hub center) and received induction therapy (steroids and basiliximab/anti-thymocyte globulin [ATG] according to donor type and immune risk) and maintenance immunosuppression mainly composed of tacrolimus (10−15 ng/ml for the first three months and of 6−8 ng/ml thereafter), mycophenolate mofetil/mycophenolic acid, and/or steroids (progressively tapered to 5 mg/day or withdrawn according to patients characteristics and immunological risk). After discharge, post-transplant care followed a standardized schedule, and every recipient was monitored by the Hub transplant center with at least one annual visit, as well as by the local nephrologist (eleven peripheral centers covering most of the Piedmont region) for periodic follow-up.

KTRs were divided into subgroups based on the characteristics of hematological disorders, including PCDs, acute leukemia, high-grade lymphoma/PTLDs, MPNs, MDS/MPN, and genetic/AA amyloidosis.

Clinical diagnosis was based on available laboratory parameters (serum electrophoresis, serum, and urinary immunofixation and light kappa and lambda chains for SMM and MM; blood count and peripheral blood smear for leukemia, MPNs, and MDS/MPN) and, if available, histopathological data (bone marrow or other tissues biopsies).

Recipients’ follow-up was obtained by scheduled clinical visits or hospital admissions when significant complications occurred. Data were collected from patients’ charts at the time of transplant and the 1st, 2nd, 5th, 10th, 15^th^ year, and last follow-up visits in our post-transplant outpatient unit. Specific items (sex, age, underlying nephropathy, type of dialysis and its duration before KT, previous transplant or immunosuppressive therapies), data about the hematological disorder (subtype, treatments, time before kidney transplant, follow-up, and occurrence of post-transplant progression/relapse), type of transplant (single or dual KT, combined, from deceased or living donor), immunosuppressive therapy, graft function (serum creatinine, eGFR with CKD-EPI formula, and 24-hours proteinuria) were retrospectively collected. The follow-up ended on July 31st, 2023.

This study was conducted in accordance with the most recent version of the Declaration of Helsinki. The clinical and research activities being reported are consistent with the Principles of the Declaration of Istanbul as outlined in the Declaration of Istanbul on Organ Trafficking and Transplant Tourism. Our Ethical Committee approval covers this study, as per resolution 1449/2019 on 11 August 2019 (“TGT observational study”).

### 2.2 Outcomes

The primary endpoint of this study was to evaluate the effect of pre-transplant oncohematological disease on death-censored graft survival and the risk of rejection. Secondary endpoints included identifying the risk of disease recurrence, the impact on patient survival rates and graft function, and the global infection risk. We, therefore, compare the hematological cohort with a control cohort of patients matched for baseline characteristics (age at transplant, sex, type of dialysis, and graft function at transplant) who do not have a history of pre-transplant oncohematological disease.

### 2.3 Statistical analysis

Each transplant performed on patients with a previous hematological disorder was matched 1:2 for age at transplant, gender, type of dialysis, and eGFR at the time of the transplant with transplants performed on patients without a hematological disorder.

The normal distribution of continuous variables, both overall and within subgroups, was assessed using the Kolmogorov-Smirnov test.

The median, first quartile, and third quartile were used to describe continuous data.

Categorical variables were summarized as counts and proportions.

We examined confounders and correlations using the nonparametric Mann-Whitney test for continuous variables, according to their distributions, and Person’s or Fisher’s Chi-Square test for categorical variables.

Survival curves were plotted with the Kaplan–Meier method, and strata were compared using the Log-Rank test.

The significance level for the study was determined prior to data collection and was set at 0.05.

All statistical analyses were performed using SPSS (IBM Corp., Released 2023. IBM SPSS Statistics for Windows, Version 29.0.2.0, Armonk, NY: IBM Corp).

## 3 Results

### 3.1 Population characteristics at baseline and cohort analysis

Between January 1, 2000, and March 31, 2023, a total of 3019 kidney transplants were performed in 2871 patients at the Turin University Renal Transplant Center “A. Vercellone.” Among them, 30 patients (receiving 31 KTs) have a pre-existing oncohematological disease (hematological cohort): 7 (23.3%) PCDs (MM n=3, AL amyloidosis n=2, LCDD n=2), 4 (13.3%) acute leukemias, 8 (26.7%) high-grade lymphomas/PTLDs (lymphomas n=3, PTLD n=5), 4 (13.3%) MPNs (polycythemia vera n=2, chronic myeloid leukemia n=1, essential thrombocythemia n=1), 2 (6.7%) MDS/MPN (myelodysplastic/myeloproliferative neoplasm with ring sideroblasts and thrombocytosis n=1, myelodysplastic/myeloproliferative neoplasm, not otherwise specified n=1). Additionally, five patients (16.7%) have a history of AA or familial amyloidosis and were described separately.

**Figure 1:**
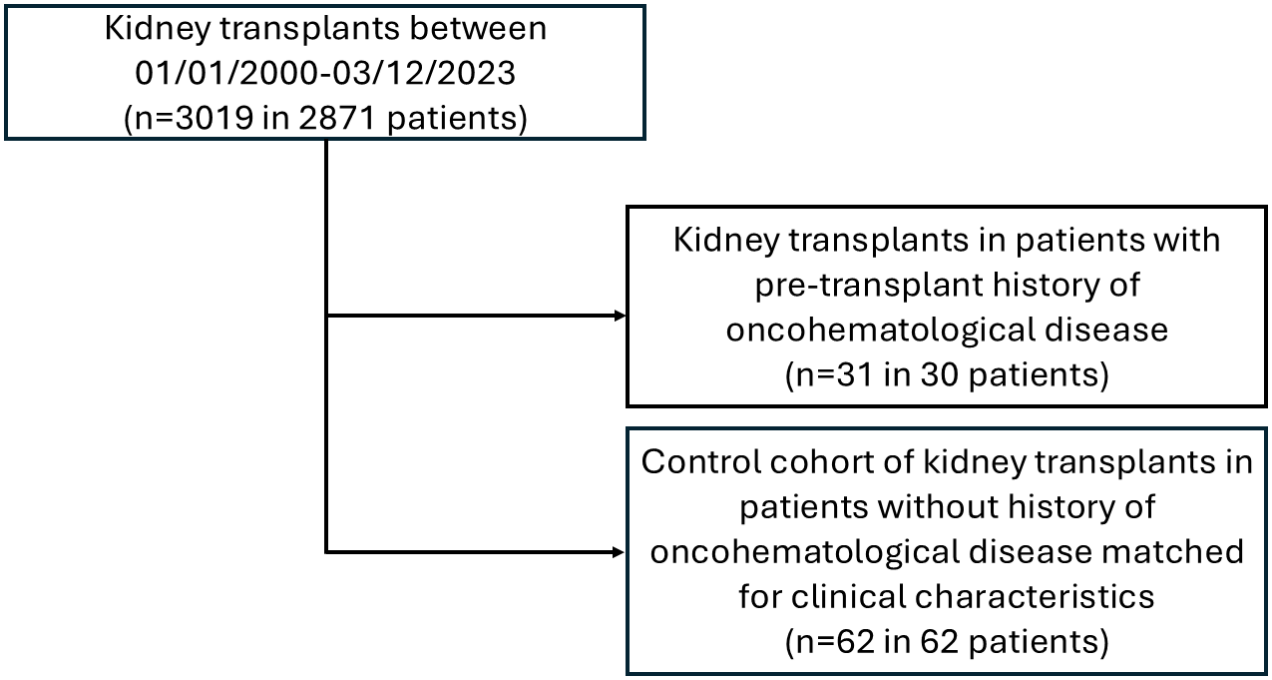
Flow chart and graphical schematization of the studied population.

The control cohort includes 62 KTs performed at our Center between 2003 and 2023 (**Figure 1**). Regarding baseline characteristics, KTs in patients with a pre-existing oncohematological disease and the control group have similar M/F ratio, age at KT, percentage of patients on hemodialysis or peritoneal dialysis before KT, and immunosuppressive treatments for both induction and maintenance regimens (**Table 1**).

**Table 1:** Baseline characteristics of the studied population.

Characteristics	KTs in patients with pre-existing oncohematological disease (n=31)	Control cohort (n=62)	p
Men/Women, n (%)	16 (51.6)/15 (48.4)	32 (51.6)/32 (48.4)	1.00
Age at KT, median (25–75 percentile), yrs	55.9 (50.6-62.3)	55.6 (48.3-60.9)	0.596
Type of Dialysis			
HD, n (%)	26 (83.8)	52 (85.2)	1.00
PD, n (%)	8 (25.8)	10 (16.4)	0.404
Immunosuppressive therapy-Induction			
ATG, n (%)	16 (51.6)	28 (49.1)	0.135
Basiliximab, n (%)	20 (64.5)	46 (80.7)	
Steroids only, n (%)	3 (9.6)	0	
Immunosuppressive therapy-Maintenance			
CNI, n (%)	26 (83.9)	57 (98.3)	0.260
MMF, n (%)	21 (67.7)	47 (81)	
AZA, n (%)	1 (3.2)	1 (1.7)	
mTORi, n (%)	6 (19.4)	11 (17.7)	
Steroids, n (%)	31 (100)	56 (96.6)	
No therapy, n (%)	3 (9.6)	0	
eGFR at transplant, median (25–75 percentile), mL/min/1.73m^2^	46 (33.8-63)	41 (28.3-45.9)	0.155
Proteinuria at transplant, median (25–75 percentile), gr/day (median)	0.4 (0.3-0.6)	0.4 (0.2-0.8)	0.801

KT, Kidney transplant; HD, hemodialysis; PD, peritoneal dialysis; ATG, Anti-thymocyte globulin; CNI, calcineurin inhibitors; MMF, Mycophenolate mofetil; AZA, Azathioprine; mTORi, mammalian target of rapamycin inhibitors.

**Figure 2:**
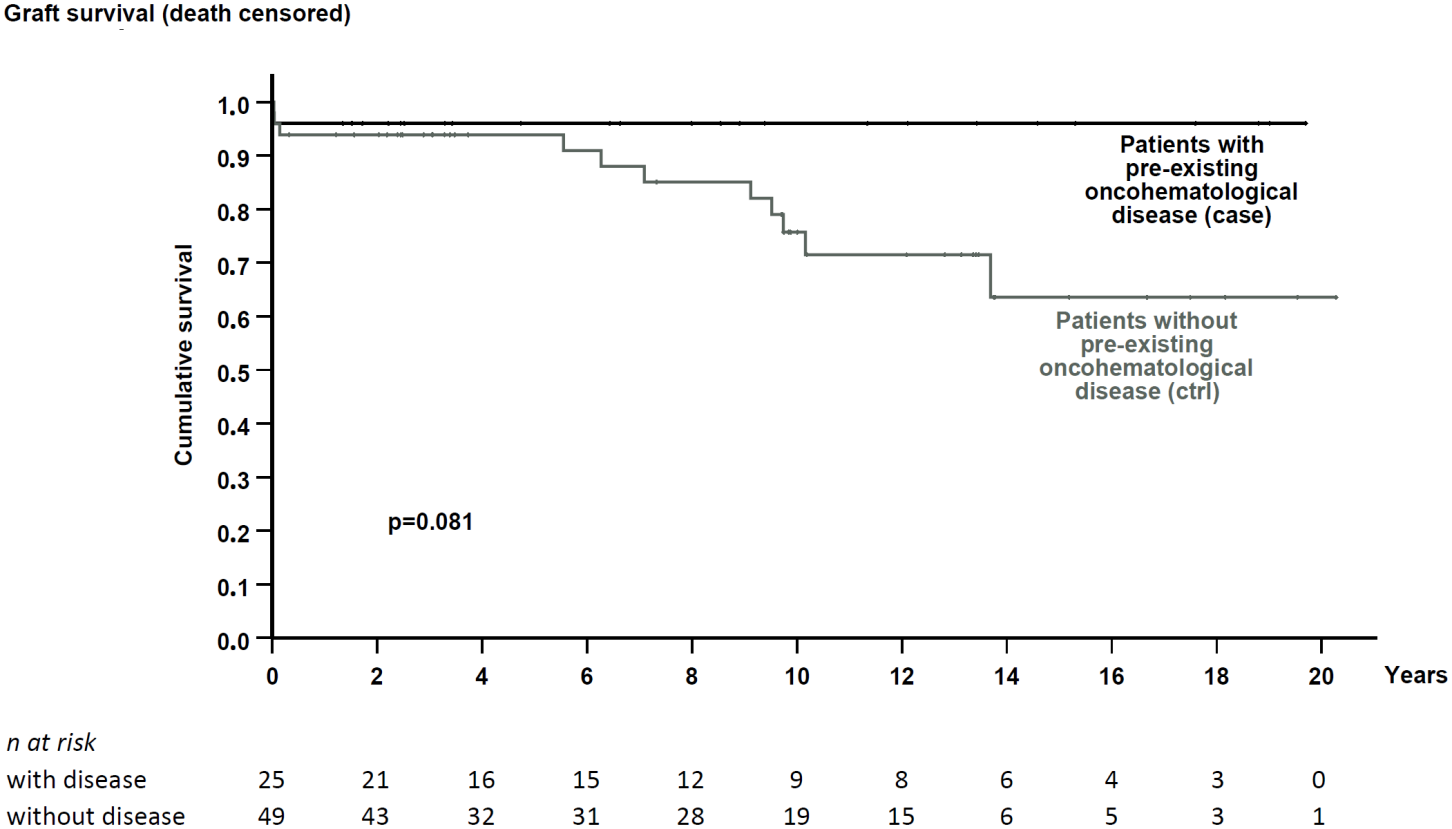
Death-censored graft survival in patients with pre-existing hematological disease and the matched control cohort. No significant difference in graft survival was noted (p = 0.081).

**Figure 3:**
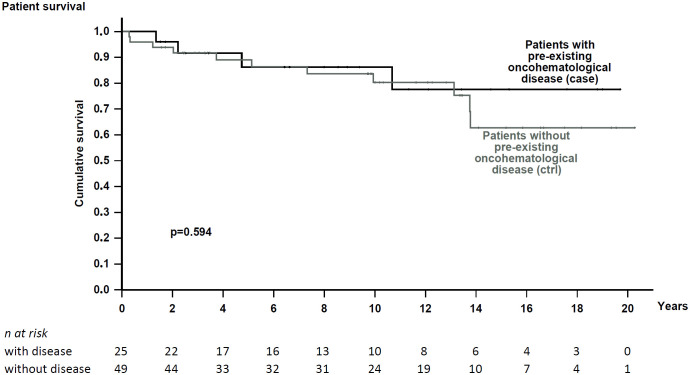
Patient survival from KTRs in patients with pre-existing hematological disease and the matched control cohort. No significant difference in survival was noted (p = 0.594).

Kidney functional data were also similar between cohorts (**Tables 1**, **2**). Patients were followed for a median time of 7.2 and 9.7 years, respectively. During this period, both exhibited similar renal function (**Table 2**) and comparable death-censored graft and patient survival rates (analyzed in patients at their first transplant; **Figures 2** and **3**).

**Table 2:** Kidney functional data, complications, and use of mTORi during the follow-up.

Characteristics	KTs in patients with pre-existing oncohematological disease (n=31)	Control cohort (n=62)	p
Follow-up, median (25–75 percentile), yrs	7.2 (2.5-11.9)	9.7 (2.5-12.8)	0.524
eGFR, median (25–75 percentile), mL/min/1.73m^2^			
One year	48 (37-67.4)	48.4 (38-62.8)	0.888
Two years	55.5 (44-65.4)	50.3 (40.4-62)	0.364
Five years	51 (41.8-65)	48.1 (37.2-57)	0.335
Ten years	46 (44.1-61.3)	48.4 (34.2-62.4)	0.872
Biopsy-proven allograft rejection, n (%)	3 (9.7)	5 (8.1)	0.732
T-cell mediated rejection, n (%)	2 (6.5)	3 (4.8)	
Antibody-mediated rejection, n (%)	1 (3.2)	1 (1.6)	
Mixed rejection, n (%)	0 (0)	1 (1.6)	
Infectious complications, n (%)	23 (74.2)	42 (67.7)	0.637
BK-DNA positive viral load, n (%)	1 (3.3)	4 (6.5)	0.224
BK nephropathy, n (%)	0 (0)	0 (0)	1.00
Post-KT neoplasia, n (%)	5 (16.1)	12 (19.4)	0.782
mTORi during follow-up, n (%)	6 (19.4)	11 (17.7)	1.00

Post-transplant neoplasia rates are also similar between groups (16.1% in the hematological cohort and 19.4% in the control cohort, p = 0.782); only two patients experienced hematological disease recurrence, specifically a light-chain deposition disease and a Castleman disease, which are discussed separately below.

Both cohorts experienced various infection episodes; however, despite no statistically significant difference between the two groups, the hematological cohort showed a reduced incidence rate of CMV infection compared to the controls (9.7% vs. 22.6%, p < 0.005).

Hematological and control cohorts showed similar rejection rates (9.7% vs. 8.1%, respectively; p = 0.732; details regarding rejection subtypes and BANFF scores are included in **Supplementary Table S1** in the **Supplementary Materials**). Although not statistically significant, only 3 out of 31 individuals (9.7%) in the hematological cohort developed *de novo* anti-HLA donor-specific antibodies (DSAs), compared to 9 out of 62 in the control group (14.5%). Notably, none of the KTRs in the hematological cohort with positive DSA exhibited clinical symptoms of antibody-mediated rejection [one patient developed a suspicious AMR with glomerulitis, but the DSA was negative, and the condition resolved after therapy with intravenous immunoglobulin (**Supplementary Table S1**)]. The eGFR for each group of DSA-positive patients is included in **Supplementary Table S2**. As expected, patients in both groups experienced a progressive decline in eGFR from baseline (defined as the time of the first DSA detection), with no differences between the two cohorts.

### 3.2 Detailed analysis of the studied patients according to their pre-transplant hematological disease

Data about all studied patients with a history of pre-transplant hematological conditions are included in **Table 3**. Additional available information is contained in the **Supplementary Material**. Briefly, only two subjects experienced disease recurrence: one with light-chain deposition disease 14 months after KT, who was successfully treated with chemotherapy (bortezomib and dexamethasone), and one with Castleman disease four years after KT, despite treatment with chemotherapy (cyclophosphamide plus steroids), achieving a complete remission. Additionally, three patients received living transplantation from the same donor of the previous bone marrow transplant and were treated exclusively with steroids for induction and maintenance therapy (immunosuppressive therapy was definitively stopped within the first year). We therefore reevaluated our results, excluding these patients, to highlight the potential impact on outcomes based on their different immunosuppressive approaches. However, this analysis yielded superimposable results, with no differences in patient and graft survival (**Supplementary Tables S3**, **S4** and **Supplementary Figures S1**, **S2** in **Supplementary Materials**).

**Table 3:** Detailed description of patients with pre-transplant oncohematological disease and outcomes after kidney transplant.

Sex	Pre-transplant oncohematological disease	Treatment for oncohematological disease	Outcome after treatment	Period before complete/stable remission and transplant	Age at transplant	Disease relapse/ recurrence	Follow-up (yrs)	Actual status
M	MM	VAD + HDCTX/ASCT + melphalan+ thalidomide	CR	9 yrs	66	None	8.9	Alive, funcioning graft
M	SM	None	NA	1 yrs and 7 months*	51	None	6.6	Alive, funcioning graft
M	SM	None	NA	9 yrs	63	None	1.5	Alive, functioning graft
F	AL	melphalan+ dexamethasone; dexamethasone + thalidomide; bortezomib+ dexamethasone	CR	2 yrs and 9 months	65	None	14.6	Alive, funcioning graft
F	AL	cytarabine+RTX; cytarabine+RTX+ASCT; melphalan +ASCT (2 times); RTX	CR	3 yrs and 8 months	54	None	18.8	Alive, functioning graft
F	LCDD	melphalan+ dexamethasone + thalidomide; thalidomide, cyclophosphamide +dexamethasone bortezomib+ dexamethasone	CR	2 yrs and 6 months	44	Yes (successfully treated with bortezomib + dexamethasone)	13.4	Alive, functioning graft
F	LCDD	cytarabine; alkeran+ASCT; melphalan+ dexamethasone + thalidomide	CR	3 yrs and 6 months	57	None	15.3	Alive, functioning graft
M	AML	CHT+ cyclophosphamide+TBI; HSCT	CR	21 yrs	36	None	9.4	Alive, functioning graft**
M	AML	CHT+RT; HSCT	CR	11 yrs and 8 months	64	None	6.4	Alive, functioning graft**
M	ALL	prednisone, vincristine, daunorubicine, asparaginase + MTX (lumbar puncture), cytarabine, methylprednisolone; cytarabine+etoposide; cytarabine+idarubicin	CR	12 yr	29	None	11.3	Alive, functioning graft
F	APL	all-trans retinoic acid + idarubicin+ cytarabine	CR	6 yrs	33	None	3.4	Alive, functioning graft
F	NHL	Partial gastrectomy+RT	CR	19 yrs	59	None	1.3	Death with functioning graft
M	NHL	R-CHOP	CR	5 yrs and 2 months	55	None	3.3	Alive, functioning graft
F	HL	CHT+RT	CR	28 yrs	41	None	2.5	Alive, functioning graft
M	PTLD	RTX+RT+mechlorethamine	CR	14 yrs and 8 months	55	None	7.7	Alive, functioning graft
F	PTLD	RTX+R-CEOP+graft nephrectomy	CR	10 yrs	30	None	8.5	Alive, failed graft
M	PTLD	Reduction of immunosuppressive therapy and graft nephrectomy	CR	9 yrs	51	None	0	Alive, failed graft (primary non-function)
F	PTLD	Reduction of immunosuppressive therapy	Stable disease without progression	5 yrs	66	None	9.7	Alive, functioning graft
M	PTLD	RTX+R-CHOP	CR	15 yrs and 5 months	62	None	1.2	Alive, functioning graft
M	CML	busulfan + cyclophosphamide; HSCT	CR	7 months	38	None	19	Alive, functioning graft**
F	Myeloproliferative neoplasm (Polycythemia vera)	None	Stable disease without progression	7 yrs*	55	None	0	Alive, failed graft (primary non-function)
				10 yrs*	58		6.7	Alive, functioning graft (2^nd^ transplant)
M	Myeloproliferative neoplasm (Polycythemia vera)	Phlebotomy+oral anticoagulant	Stable disease without progression	10 yrs*	60	None	8.6	Alive, functioning graft
F	Myeloproliferative neoplasm (Essential thrombocythemia)	None	Stable disease without progression	13 yrs*	50	None	1.7	Alive, functioning graft
F	Myelodysplastic/ myeloproliferative neoplasm with ring sideroblasts and thrombocytosis	None	Stable disease without progression	1 yrs and 3 months*	64	None	19.7	Alive, functioning graft
M	Myelodysplastic/ myeloproliferative neoplasm, not otherwise specified	None	Stable disease without progression	6 yrs*	67	None	2.5	Alive, functioning graft
M	AA	None	Stable disease without progression	7 yrs*	58	None	8	Alive, functioning graft
F	AA	Surgical removal of abdominal lesion	CR	4 yrs	57	None	4.7	Death with functioning graft
M	AA	None	Stable disease without progression	3 yrs*	50	None	2.2	Death with functioning graft
F	AA	Surgical removal of abdominal lesion	CR	3 yrs	56	Yes (successfully treated with cyclophosphamide + dexamethasone)	17.6	Alive, functioning graft
F	AA	infliximab; etanercept; adalimumab+steroids	CR	2 yrs and 2 months	52	None	12.1	Alive, functioning graft

*Time between diagnosis and transplant.

**Kidney transplant from the same living donor of HSCT (no need for prolonged immunosuppressive therapy).

MM, multiple myeloma; SM, smoldering myeloma; VAD, vincristine, doxorubicin, dexamethasone; HDCTx/ASCT, High-dose chemotherapy followed by autologous stem cell transplantation; AL, Amyloid light-chain; LCDD, light chain deposition disease; RTX, rituximab; NHL, Non-Hodgkin Lymphoma; HL, R-CHOP: rituximab, cyclophosphamide, doxorubicin, vincristine, prednisolone; CHT, chemotherapy; CML, chronic myeloid leukemia; HSCT, Hematopoietic stem-cell transplantation; AML, acute myeloid leukemia; TBI, total body irradiation; ALL, acute lymphoblastic leukemia; APL, acute promyelocytic leukemia; MTX, methotrexate; PTLD, post-transplant lymphoproliferative disease; R-CEOP, rituximab, cyclophosphamide, etoposide, vincristine, prednisolone.

## 4 Discussion

Oncohematological disorders include various diseases with different characteristics, ranging from benign to life-threatening. The incidence in the general population and the age at presentation of patients vary dramatically according to the type of disease (10).

For example, MM usually occurs in older adults (median age at diagnosis of 66 years with only 10% <50 years) and accounts for approximately 1% of malignant diseases and 10-13% of all hematologic malignancies (10–12); AL amyloidosis is an uncommon disorder (incidence of approximately 9 to 14 cases per million person-years in the United States) with a median age at diagnosis of 64 years and less than 5% of patients under the age of 40 (13, 14); leukemia and lymphoma can occur both in young adult and in older patients depending on the subtypes of disease (15).

According to these data, it is not surprising that, considering the prolonged life expectancy, all these disorders may represent a significant problem in pre-transplant evaluation.

In this context, several key questions need to be addressed: the risk of disease progression or recurrence after KT and the potential role of previous hematological disease in influencing patient and graft survival, including post-transplant complication rates and rejection risk. KDIGO 2020 (5), the most updated international guideline, underlines that “decisions about kidney transplantation in patients with a prior history of hematologic malignancy who are now in remission should be made in collaboration with a hematologist with transplant experience in determining transplant candidacy, since many lesions may be deemed to be at high risk of accelerated progression or transformation post-transplant.” This is unsurprising, considering that the available literature on these patients primarily derives from case reports or series, which are often limited to a specific condition or disease.

This paper reports our experience with all 30 KTRs with a pre-existing hematological disease who underwent 31 KTs between 2000 and 2023.

Few studies have specifically evaluated the risk of rejection in this population. An association between lenalidomide and increased allograft rejection due to direct immunomodulatory effect has been proposed in patients with MM concomitant with functioning KT (16). Still, no specific association between rejection and other previous or concomitant MM therapy has been reported. In Ruphael et al. (17), three out of 8 retransplanted PTLDs experienced acute or chronic rejection; this percentage is lower in Johnson et al. (18), where maintenance immunosuppression between the first and second transplant is almost superimposable. In other subsets, Leung et al. (19) reported acute rejections in 3 of 7 patients with a history of light chain disease. Despite these data being confirmed in a larger cohort, only three acute rejection episodes were recorded, and three patients developed *de-novo* DSAs without clinical signs of antibody-mediated rejection. As expressed by other authors (18), a tailored approach with specific attention to pre-transplant disease and patient characteristics allows us to maintain adequate immunosuppression, avoiding rejection while minimizing the risk of recurrence.

Considering recurrence rates, only two KTRs have a disease relapse of their pre-transplant hematological disease, one with LCDD and one with Castleman disease.

Aggressive disease management to achieve complete remission appears crucial in the pre-transplant context. A high rate of LCDD recurrence is reported in the literature. Leung et al. observed recurrent LCDD in 5 of 7 transplanted patients after a median time of 33.3 months (19). Various case reports confirm these findings (20–24). Our patient had undergone multiple subsequent chemotherapy treatments before KT for disease recurrences after achieving the first remission, and the transplant was performed three years later. Despite this approach, one year after KT, a significant increase in serum light chain was noted, associated with a slight rise in serum creatinine. The patient was treated with steroids and bortezomib with a partial response; subsequent new serum light chains increased, requiring other lines of therapy, finally achieving a complete remission with no other evidence of recurrence within the following four years.

The second recurrence was observed in a patient with Castleman disease who underwent KT 3 years after achieving complete remission. The event occurred four years after the transplant as a multicentric presentation. It was treated with steroids and cyclophosphamide with full recovery (no evidence of hematological relapse at the last visit, eight years after recurrence). Limited data are available in the Literature for KT with a pre-existent Castleman disease: Murakami reported a good post-transplant clinical course (follow-up eight years) in a patient with pre-transplant multicentric Castleman disease (25), while Yousif described a case of monocentric Castleman disease incidentally diagnosed during KT with normal graft function and no disease recurrence during the follow-up (26).

Regarding the other oncohematological diseases, although MM has been considered for a long time as a contraindication for KT due to the increased risk of graft failure, severe and life-threatening infections, and recurrence (19, 24, 27–29) nowadays, literature reports favorable results in patients who achieved disease remission (30, 31), primarily when KT is performed from the same donor of bone marrow transplantation (32–36) despite, as also specified in KDIGO (5), no indication about the wait time between remission and transplantation is even available.

In the case of smoldering myeloma, guidelines now suggest not excluding candidates from kidney transplantation, despite a significant risk of transformation into multiple myeloma (not precisely quantifiable), should be considered and appropriately discussed (5). Despite having a limited number of patients and not being treated with recently available drugs that have further modified the approach to the disease, even hypothesizing a potential transplant in patients under chemotherapy in stable disease, the results in our patients confirm all these findings.

Furthermore, similarly to Literature data (37, 38), in our study, no patient with a pre-transplant history of leukemia or lymphoma/PTLD had disease recurrence after transplant. In three patients who had received KT from the same living donor after a previous bone marrow transplant, it was also possible to definitively stop immunosuppressive therapies, resulting in a remarkable overall patient and graft survival. The feasibility of re-transplantation in patients with previous post-transplant lymphoproliferative disorders is described in the literature, with favorable clinical outcomes and no disease recurrence (17, 18, 39–42). This is also evident in our KTRs, where no recurrence was observed in any of the four patients. International guidelines depicted a variegated approach in these patients: KDIGO (5) remarks to avoid transplanting patients with leukemia or lymphoma until they have received curative therapy, achieved remission, and remained cancer-free for a period to be determined in consultation with the patient, a hematologist/oncologist, and the transplant program; in contrast, other guidelines suggest a definite period (2 years for Canadian Society of Transplantation [1], Kidney Health Australia-Caring for Australasians with Renal Impairment [2] and American Society of Transplantation guidelines [3] and 1–3 years for European Renal Best Practice [4]) before a patient could be considered for KT. Considering that some drugs (e.g., tyrosine kinase inhibitors) have completely transformed the life expectancy of these patients despite maintenance therapy (43), this approach may also change shortly.

The need for a dedicated and expert hematological consultation is even more critical in patients with a history of myelodysplastic or myelodysplastic/myeloproliferative neoplasms, for which recommendations are significantly lacking, and most reported cases included patients with chronic myeloid leukemia treated with imatinib (43, 44). None of the patients in both groups, which is a remarkable cohort based on scarce literature data, developed disease relapse or recurrence after KT.

We are also aware that some studies have shown unsatisfactory results in patients with a history of pre-transplant malignancies. The analysis of the UNOS database in 2019 by Livingston-Rosanoff et al. (45) reported that pre-transplant malignancies are progressively increasing in number across the US, but it is associated with an increased risk of post-transplant malignancies, graft loss, and decreased overall survival. Non-melanoma skin cancer was the most common diagnosis for patients with and without pre-transplant malignancies (66.2% vs 57.1%), followed by lung cancer (5.2% vs 6.6%). The post-transplant malignancies of 228 individuals with pre-transplant malignancies were classified as recurrences of their original pre-transplant malignancies by UNOS, representing a 2% recurrence rate in patients with pre-transplant malignancies. Of the patients who experienced recurrence, the majority (48%) were solid organ cancers, followed by non-melanoma skin cancer (21%), unknown (12%), and melanomas (7%). Hematopoietic recurrences are described, although they are relatively uncommon (12%), and are primarily associated with leukemia, lymphomas, and other myelodysplastic disorders. The authors emphasized in their conclusions the importance of collaborative database development between transplant and cancer registries to better define the interrelationship between pre-transplantation and cancer survivorship versus freedom from prolonged dialysis, an issue that is currently underestimated (46).

In the German paired analysis by Becker et al. (47), KT recipients with a history of pre-transplant malignancy had lower five-year death-censored as well as overall graft survival. Cox proportional hazard modeling showed a correlation between pre-transplant malignancy and inferior graft survival; however, among the 65 KT recipients studied, only one patient had a hematologic malignancy.

Serkies et al. (48) recently proposed a review and discussion of malignancies in adult kidney transplant candidates and recipients updated to 2023. Albeit the primary focus are solid neoplasia, they also reported that, based on all available data, along with changing patient characteristics and the availability of newer cancer therapies, a shorter waiting time to determine suitability for transplant in pre-transplant malignancies patients could be appropriate for cancers with substantially improved survival in the general population, including multiple myeloma cases with a complete remission after successful treatment with preconditioning chemotherapy followed by high-dose alkylating agents and autologous stem cell transplant. They also clearly stated that transplant suitability and waiting time for candidates with cancer should be individualized, with the decision to consider transplantation made by a multidisciplinary team involving oncologists/hematologists, transplant nephrologists, patients, and their caregivers. Expected survival and quality of life on dialysis versus transplantation, projected cancer recurrence risk, including the effect of administered immunosuppression, estimated survival depending on tumor type, and, given current treatment possibilities, if recurrence post-transplant occurred, should be considered. Of note, prolonged dialysis is associated with an increased risk of complications, including malignancies and death.

We suggest that our analysis, focuses on a specific settings with poor literature data and many diseases with uncommon incidence and recent reclassification, offers the opportunity to improve the available information stressing the importance of an appropriate and in-depth evaluation of these patients that are at high risk to be excluded for transplant, and at the same time that the multidisciplinary approach with hematologist trained in these condition (as also expressed by the interntational guidelines) is crucial.

Our study has several limitations, including the relatively small sample size and the lack of routine protocol biopsies, which may have underestimated graft damage due to disease relapse in some conditions, particularly in the early stages. On the other hand, to the best of our knowledge, this is the first experience that analyzed characteristics and outcomes of patients with pre-existing oncohematological diseases in comparison to a control cohort with similar features and post-transplant management, showing positive results (especially regarding rejection risk and disease recurrence) and no significant differences in clinical outcomes. These positive results may be partially derived from a homogeneous, tailored, and multi-disciplinary management, with particular attention to the immunosuppressive therapy, avoiding the risk of an excessive “pressure” in patients with potential risk of relapse/recurrence for one side and an excessive underimmunosuppression for the other; the difference in CMV prevalence vs. the control cohort matched with a very low-incidence of acute rejection and *de-novo* DSA corroborated this strategy.

In conclusion, based on our findings and Literature data, we suggest that patients with pre-existing oncohematological disorders should not be denied KT after a well-documented stable disease. In this context, a multidisciplinary approach is crucial for establishing standardized pre- and post-transplant monitoring protocols and achieving optimal graft and patient outcomes.

Extensive registry-based studies are needed to support our findings, especially for rare conditions such as MPNs, MDS/MPN, and amyloidosis.

## Data Availability

The original contributions presented in the study are included in the article/**Supplementary Material**. Further inquiries can be directed to the corresponding author.
