# Supplementary material for: Pre-existing oncohematological disease in kidney transplant recipients: impact on graft survival, acute rejection, and long-term clinical outcomes
Source: Front Immunol. 2025 Aug 6;16:1629521. doi: 10.3389/fimmu.2025.1629521 (PMC12364700; doi:10.3389/fimmu.2025.1629521)

Supplementary Material

# Supplementary clinical data

***Plasma cell dyscrasias (PCDs)***

The PCD group included seven kidney transplant recipients (KTRs).

The multiple myelomas (MMs) were three male patients. All were on hemodialysis before KT for ten years, one year, and almost six years, respectively; one had undergone a heart transplant before KT.

Two out of three patients have been classified as smoldering myeloma (SMM) with CKD due to tubulointerstitial disease and underwent a "watchful waiting" strategy; the other is an MM with associated AL amyloidosis and received chemotherapy with subsequent autologous bone marrow transplant, achieving complete remission.

In pre-transplant management, the patients with smoldering myeloma were evaluated with bone marrow biopsy, showing a stable disease in both cases (time between BOM and KT was respectively 19 months and 9 years) and a persistent complete remission in the other case; time between remission and KT was 9 years.

They underwent SKT from a cadaveric donor. The immunosuppressive regimen consisted of basiliximab and steroids in two cases, ATG, mycophenolate mofetil (MMF), and steroids in one (induction therapy), and CNI, MMF, and steroids as maintenance therapy. Eculizumab was used as induction and maintenance therapy in 1/3 of patients for aHUS.

All patients are still alive; 1/3 experienced a SARS-CoV-2 infection without hospitalization and also had a post-transplant cutaneous neoplasm, but all resolved with complete recovery.

One patient had evidence of suspicious (borderline) acute T cell-mediated rejection treated with steroids, with complete remission and cryocrystaglobulinemia without needing therapy and stable graft function. None had a relapse of the hematological disease.

Patients with pre-existing AL amyloidosis were two, one male and one female, with ages at KT of 54 and 65 years, respectively. Both were on hemodialysis for a median time of 3.5 years (3-4) due to the underlying hematological disorder; one patient also had a gastrointestinal involvement. Both subjects achieved complete remission before KTs (median time, 3 years and 3 months) after chemotherapy plus autologous stem cell transplantation (ASCT) in the first case and two chemotherapy regimens (one administered after disease relapse) in the second subject. Both patients received a single kidney transplant from a cadaveric donor with basiliximab plus steroids at induction, with a maintenance therapy composed of an mTOR inhibitor, MMF, and steroids in KTRs who received ASCT. At the same time, the remaining one was treated with "standard" therapy (CNI-MMF-Steroids).

Both patients had a functioning graft at the end of follow-up. No allograft rejection was recorded.

One patient experienced a lung infectious episode and post-transplant cutaneous neoplasia (all resolved with complete recovery); the other patient had liver disease associated with HCV before their transplantation and was already undergoing treatment with direct-acting antivirals (DAAs). The patient was reevaluated for HCV RNA negativity two years after the transplantation.

The two female patients with a history of CKD due to light-chain deposition disease (kappa light chain in both cases) received a single kidney transplant after achieving complete remission of the disease (the time between remission and KT was 32 months and 43 months, respectively).

Both underwent chemotherapy (with the addition of ASCT in one) for hematological disease treatment. Induction was composed of basiliximab and steroids, and maintenance therapy with CNI and steroids (with associated MMF in one). No one documented acute rejections. Disease relapse was observed 14 months after KTR in one patient and was successfully treated with chemotherapy (bortezomib and dexamethasone).

## *Leukemia group*

The leukemia group includes three males and one female.

All were at their first transplant and have been treated with steroids or immunosuppressive therapies before KT. All patients were on dialysis before KT. Underlying nephropathy was unknown in half of the patients; 2/4 had CKD due to tubulointerstitial disease.

Two patients had acute myeloid leukemia, one acute promyelocytic leukemia, and one had acute lymphoblastic leukemia. All received chemotherapy (radiotherapy was performed on only one patient); 2/4 underwent an allogeneic bone marrow transplant from an HLA-identical donor with subsequent complete chimerism. Complete remission was achieved in all cases; the median time between remission and KT was 11.8 years (range, 10.3-14.3 years).

Only two patients underwent SKT from a cadaveric donor (basiliximab plus steroids and ATG plus MMF and steroids, as induction; CNI-MMF-Steroids and CNI plus steroids as maintenance therapy); two out of 4 patients received living transplantation from the same donor of the previous bone marrow transplant and were treated exclusively with steroids for induction and maintenance therapy (immunosuppressive therapy was definitively stopped within the first year).

All patients were alive and had a functioning graft at the time of the last visit. No patient experienced leukemia relapses; one out of four had post-transplant solid neoplasia (thyroid neoplasia), all of which resolved with complete recovery.

## *High-grade lymphoma/PTLD group*

Three patients had a pre-transplant history of lymphoma: two females and one male with gastric non-Hodgkin lymphoma (NHL), Hodgkin lymphoma (HL), and one with Diffuse large B-cell lymphoma (DLBCL), respectively. All patients achieved complete remission. The first patient had a partial gastrectomy and radiotherapy; the second received radiotherapy and chemotherapy; the third only chemotherapy. Two-thirds were on dialysis before kidney transplant (one undergoing hemodialysis and one undergoing peritoneal dialysis). The median duration from remission to kidney transplantation was 5.1 years and 19 years, respectively.

CKD was due to polycystic kidney disease in 1/3, chronic glomerulonephritis in 1/3, and multifactorial in 1/3.

Two-thirds of the patients underwent single kidney transplants from cadaveric donors (one was a combined kidney-liver) and one from a living donor. The immunosuppressive regimen for induction varied among patients: basiliximab plus steroids, basiliximab plus MMF and steroids, and ATG plus MMF and steroids. The maintenance therapy was the same for all: CNI, MMF, and steroids.

One patient died of a heart attack 11 months after KT with a functioning graft without documented lymphoma relapse during her follow-up.

Five KTRs (3 men and two women) had a previous history of PTLD. All of them were on dialysis before the second KT. CKD was due to polycystic kidney disease in 1/5, glomerulonephritis in 1/5, kidney malformation in 1/5, glomerulonephritis plus Alport syndrome in 1/5, and not known in 1/5. One patient had B-cell chronic lymphatic leukemia, three had large B-cell lymphomas, and one had plasmacytoma-like PTLD; 4/5 had exclusive graft localization and 1/5 had gastric localization only; the disease was EBV-related in 2/5 cases.

The first patient underwent immunosuppression reduction with no evidence of disease progression. The other patients were treated with anti-CD20 rituximab (2/5), chemotherapy (2/5), radiotherapy (1/5), high-dose steroids (1/5), or graft removal (1/5). One patient had a disease relapse treated with rituximab. All patients achieved complete and persistent remission, allowing them to undergo a new kidney transplant (KTR) after a median time of 10 years (range, 7-15).

All patients underwent a single kidney transplant from a cadaveric donor. Regarding the immunosuppressive regimen, basiliximab was used in three of five patients, and ATG in two of five patients. Three of five patients were treated with CNI, MMF/MPA, and steroid maintenance therapy, and two of five with mTORi instead of MMF.

One patient had suspicious AMR on a protocol kidney biopsy (evidence of isolated glomerulitis without DSA or C4d positive), which was treated only with endovenous immunoglobulins (due to his high infection risk), with recovery.

One patient immediately lost their graft due to arterial thrombosis; one patient had a pulmonary infectious complication; one had a paucisymptomatic COVID-19 disease with complete recovery; one developed a squamous cell carcinoma. None had PTLD recurrence.

***Myeloproliferative neoplasms***

Four patients have a pre-transplant documented myeloproliferative neoplasm (two cases of essential thrombocythemia, one case of polycythemia vera, and one case of chronic myeloid leukemia). Only the patient with polycythemia vera had previously undergone another KT, who immediately lost after reperfusion due to acute rejection. The male patients with chronic myeloid leukemia received busulfan +

cyclophosphamide and allogeneic bone marrow transplant, achieving complete remission. The other patients have stable disease at the time of KT. The median time from remission or stabilization of the disease to KT was 8.5 years (5.4-8.1).

Two patients underwent a single kidney transplant from a cadaveric donor, and one from a living donor; 1/4 underwent SKT from the same living donor of the bone marrow transplant and received only steroid induction (tapered and suspended at one-month post-transplant). The immunosuppressive regimen for the other patients included Basiliximab (1/3 plus ATG) and CNI, MMF, and steroids as maintenance therapy.

Three out of four patients had a functioning graft at the end of the follow-up: one patient died of pulmonary SARS-CoV-2-related infection with a functional graft.

One patient had a relapse of nephropathic disease (extra proliferative glomerulonephritis p-ANCA positive) and an associated acute T-cell mediated rejection, and was treated with steroids and rituximab with subsequent recovery.

No progression or recurrence of the hematological disease was recorded.

***Myelodysplastic/myeloproliferative neoplasms***

Two patients have a pre-transplant documented myelodysplastic/myeloproliferative neoplasm (one with myelodysplastic/myeloproliferative neoplasm with ring sideroblasts and thrombocytosis, and one with myelodysplastic/myeloproliferative neoplasm, not otherwise specified). Both patients were not treated with immunosuppressive medication before transplant, and received transplant 1.4 and six years after documented stable disease, respectively.

Both received Basiliximab and steroids as induction, followed by CNI, MMF, and steroids as maintenance therapy, and have functioning grafts at the last follow-up.

No progression of the hematological disease was documented.

***Amyloidosis***

The amyloidosis group comprises five patients (three women and two men) with a median age at KT of 56 years (range, 50-58). Three out of five patients had been previously treated with steroids and/or immunosuppressive therapies. Four out of five were on dialysis, all undergoing hemodialysis.

Four patients had AA amyloidosis (2/4 due to Castleman disease), and 1/5 had a lysozyme gene mutation. In all cases, CKD was due to the underlying hematological disorder, and 2/4 of patients had a multiorgan disease with gastrointestinal and hepatic involvement. No therapy was performed on the one patient with hereditary amyloidosis; in the other cases, various treatments were used: biologics (1/4), surgery (2/4 with Castleman disease), azathioprine plus steroids in the remaining one, and all achieved a complete remission without disease relapse. The median time before KT was 84 months (26-73).

Four patients underwent a single kidney transplant from a cadaveric donor (1/5 had a related living donor). Basiliximab was adopted as induction in 4/5, while in the last case, only steroids were used; 3/5 were treated with CNI, MMF, and steroids, 1/5 with CNI and steroids, and 1/5 with CNI and azathioprine.

Two out of 5 patients died; however, they maintained a functional renal graft until their demise. The remaining patients retained a functioning graft at their last follow-up visit.

No allograft rejection was recorded. Only one patient with Castleman disease had a post-transplant relapse four years after KT, which was treated with chemotherapy (cyclophosphamide plus steroids), achieving complete remission.

# Supplementary Tables

**Supplementary Table S1.** Detailed description of rejection cases among hematological and control cohorts

| **Sex** | **Pre-transplant oncohematological disease** | **Rejection subtype revised (BANFF 2022 classification)** | **BANFF scores, DSAs, C4d** | **Treatment** | **Oucome** |
| --- | --- | --- | --- | --- | --- |
| M | PTLD | MVI DSA-/C4d- | g1; ptc0; i0; t0; v0; DSA-; C4d- | IgEv | Recovery |
| F | Myeloproliferative neoplasm (Essential thrombocythemia) | TCMR grade IA | g0; ptc0; i2; t2; v0; C4d-, DSA -; associated extracapillary proliferation (pANCA positive) | Steroids+Rituximab (for extracapillary proliferation) | Recovery |
| M | SM | Borderline TCMR | g0; ptc0; ptc0; i1, t2; v0; DSA+; C4d neg | Steroids | Recovery |
| M | Control | Borderline TCMR/Suspicious AMR | g1; ptc0; i0, t3; v0; C4d+/-; DSA+ | Steroids | Recovery |
| F | Control | TCMR grade IIB | g0; ptc0; i1, t2; v2; DSA-; C4d- | Steroids | Recovery |
| M | Control | TCMR grade IA | g0; ptc0; i2; t2; v0; C4d-, DSA - | Steroids | Recovery |
| M | Control | TCMR grade IA | g0; ptc0; i2; t2; v0; C4d-, DSA - | Steroids | Recovery |
| F | Control | Acute AMR | g1; ptc1; i0; t0; v2; C4d+, DSA+ | IgEv+Steroids | Recovery |

MVI: microvascular inflammation; IgEv: endovenous immunoglobulin; TCMR: T-Cell mediated rejection; AMR: antibody-mediated rejection

**Supplementary Table S2.** Kidney functional data in patients who developed DSA stratified between the hematological and control cohorts.

|  | **KTs in patients with pre-existing oncohematological disease DSA+**  **(n=3)** | **Control cohort DSA+**  **(n=9)** | **p** |
| --- | --- | --- | --- |
| Serum creatinine at DSA detection, median (25-75 percentile), mg/dL | 1.6 (1.45-1.8) | 1.8 (0.95-2.2) | 0.215 |
| eGFR at DSA detection, median (25-75 percentile), mL/min/1.73m^2^ | 42.1 (38.4-45.8) | 40.6 (26.5-62) | 0.107 |
| Serum creatinine at 6 months after DSA detection, median (25-75 percentile), mg/dL | 1.4 (1.4-1.5) | 1.8 (1.4-2.1) | 0.228 |
| eGFR at 6 months after DSA detection, median (25-75 percentile), mL/min/1.73m^2^ | 42 (38.4-46) | 40.5 (26.5-62) | 0.147 |
| Serum creatinine at 12 months after DSA detection (25-75 percentile), mg/dL | 1.4 (1.3-1.5) | 1.5 (1-2.2) | 0.294 |
| eGFR at 12 months after DSA detection, median (25-75 percentile), mL/min/1.73m^2^ | 41.4 (39-40) | 39 (34-40) | 0.197 |
| Follow-up, median (25-75 percentile), yrs | 6.8 (3.4-10.1) | 7.2 (5.7-10.1) | 0.186 |

**Supplementary Table S3.** Baseline characteristics of the studied population, excluding patients who received living transplantation from the same donor as the previous bone marrow transplant.

|  | **KTs in patients with pre-existing oncohematological disease**  **(n=28)** | **Control cohort**  **(n=56)** | **p** |
| --- | --- | --- | --- |
| Men/Women, n (%) | 13 (46.4)/15 (53.6) | 26 (46.4)/30 (53.6) | 1.00 |
| Age at KT, median (25-75 percentile), yrs | 56.9 (51.6-63.5) | 55.7 (48.9-61.7) | 0.362 |
| Type of Dialysis |  |  |  |
| HD, n (%) | 24 (85.7) | 47 (83.9) | 1.00 |
| PD, n (%) | 4 (14.3) | 9 (16.1) | 0.386 |
| Immunosuppressive therapy-Induction |  |  |  |
| ATG, n (%) | 16 (57.1) | 25 (44.6) | 0.344 |
| Basiliximab, n (%) | 20 (71.4) | 43 (76.8) |  |
| Immunosuppressive therapy-Maintenance |  |  |  |
| CNI, n (%) | 26 (83.9) | 51 (91.1) | 0.732 |
| MMF, n (%) | 21 (67.7) | 41 (73.2) |  |
| AZA, n (%) | 1 (3.2) | 1 (1.8) |  |
| mTORi, n (%) | 6 (19.4) | 11 (19.6) |  |
| Steroids, n (%) | 28 (100) | 50 (89.3) |  |
| eGFR at transplant, median (25-75 percentile), mL/min/1.73m^2^ | 43.7 (33.2-62.5) | 40.4 (29.4-60.1) | 0.167 |
| Proteinuria at transplant, median (25-75 percentile), gr/day (median) | 0.4 (0.3-0.6) | 0.5 (0.2-0.7) | 0.681 |

KT: Kidney transplant; HD: hemodialysis; PD: peritoneal dialysis; ATG: Anti-thymocyte globulin; CNI: calcineurin inhibitors; MMF: Mycophenolate mofetil; AZA: Azathioprine; mTORi: mammalian target of rapamycin inhibitors

**Supplementary Table S4.** Kidney functional data, complications, and use of mTORi during the follow-up, excluding patients who received living transplantation from the same donor as the previous bone marrow transplant.

|  | **KTs in patients with pre-existing oncohematological disease**  **(n=28)** | **Control cohort**  **(n=56)** | **p** |
| --- | --- | --- | --- |
| Follow-up, median (25-75 percentile), yr | 6.7 (2.3-11.5) | 9.8 (2.4-12.8) | 0.453 |
| eGFR, median (25-75 percentile), mL/min/1.73m^2^ |  |  |  |
| One year | 47.5 (36.4-63.9) | 47.2 (35.6-62) | 0.839 |
| Two years | 52 (40-62.7) | 49 (39.2-57.8) | 0.301 |
| Five years | 48 (37.5-64.2) | 46.1 (37-56.9) | 0.685 |
| Ten years | 45.8 (43.6-61) | 47.3 (32.8-64) | 0.959 |
| Allograft rejection, n (%) | 3 (10.7) | 5 (8.9) | 0.364 |
| Infectious complications, n (%) | 22 (78.6) | 40 (72.7) | 0.764 |
| BK-DNA positive viral load, n (%) | 1 (3.5) | 3 (5.3) | 0.633 |
| BK nephropathy, n (%) | 0 (0) | 0 (0) | 1.00 |
| Post-KT neoplasia, n (%) | 4 (14.3) | 10 (18.2) | 0.782 |
| mTORi during follow-up, n (%) | 6 (21.4) | 11 (20.4) | 1.00 |

# Supplementary Figures

**Supplementary Figure 1.** Death-censored graft survival in patients with pre-existing hematological disease and the matched control cohort (excluding patients who received living transplantation from the same donor as the previous bone marrow transplant). No significant difference in graft survival was noted (p = 0.153).


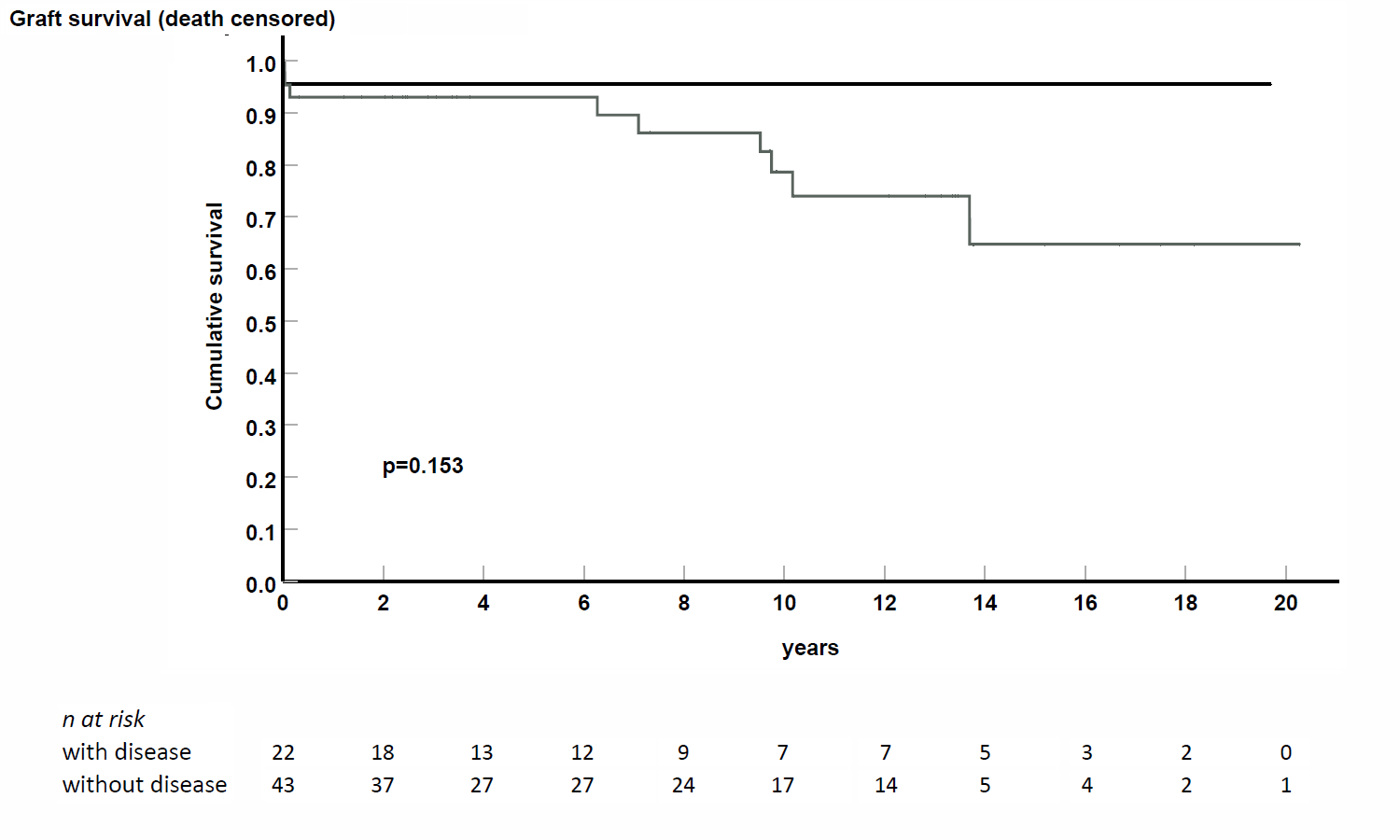


**Supplementary Figure 2.** Patient survival from KTRs in patients with pre-existing hematological disease and the matched control cohort (excluding patients who received living transplantation from the same donor as the previous bone marrow transplant). No significant difference in survival was noted (p = 0.745).


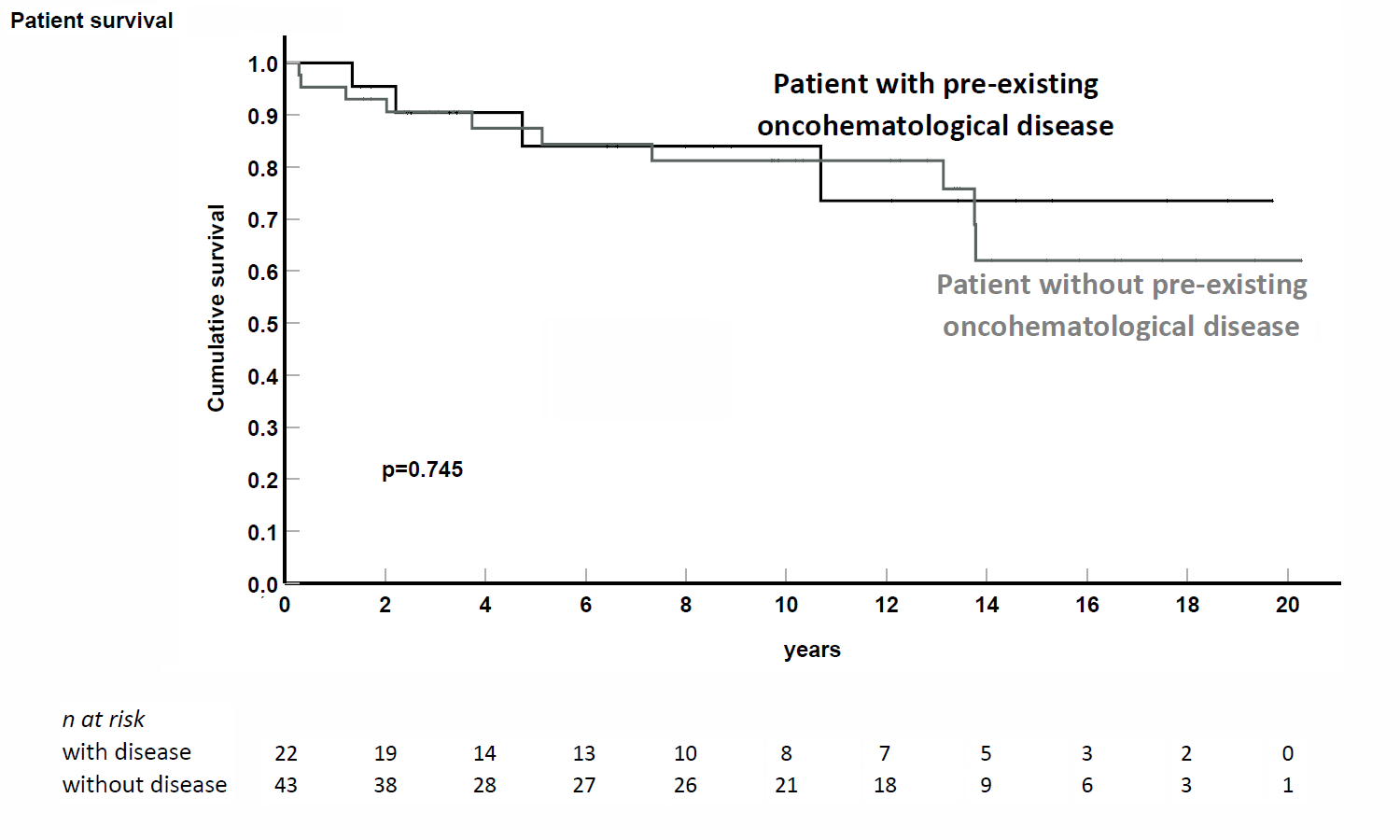

Supplement: Supplementary file 1 [file SupplementaryFile1.docx]
